# Prognostic value of elevated postoperative high-sensitivity troponin T in diabetes mellitus patients for ischemic events within 12 months after PCI in ACS patients: a retrospective cohort study

**DOI:** 10.3389/fcvm.2025.1606435

**Published:** 2025-06-27

**Authors:** Xuefei Mu, Miaohan Qiu, Shangxun Zhou, Yixuan Duan, Daoshen Liu, Kai Xu, Quanmin Jing, Yi Li, Yaling Han

**Affiliations:** ^1^ State Key Laboratory of Frigid Zone Cardiovascular Disease, Cardiovascular Research Institute and Department of Cardiology, General Hospital of Northern Theater Command, Shenyang, China; ^2^ The Department of Cardiology, Xijing Hospital, Air Force Medical University, Xi'an, China

**Keywords:** acute coronary syndrome, percutaneous coronary intervention, high-sensitivity cardiac troponin T, diabetes mellitus, ischemic events

## Abstract

**Objective:**

This study aimed to investigate the prognostic value of high-sensitivity cardiac troponin T (hs-cTnT) levels and diabetes mellitus (DM) on ischemic events within 12 months after percutaneous coronary intervention (PCI) in acute coronary syndrome(ACS) patients.

**Methods:**

This retrospective cohort study included 14,173 consecutive ACS patients undergoing PCI at the General Hospital of the Northern Theater Command between March 2016 and March 2022. The primary outcome was the occurrence of ischemic events within 12 months, defined as a composite of cardiac death, non-fatal myocardial infarction (MI), and/or stroke. Secondary outcomes included all-cause mortality at 12 months and the individual components of the primary outcome.

**Results:**

During the 12-month follow-up, the overall incidence rates of ischemic events, cardiac death, MI, stroke, and all-cause mortality were 2.19%, 1.12%, 0.58%, 0.59%, and 1.55%, respectively. Elevated hs-cTnT levels were significantly associated with increased risks of ischemic events (adjusted HR: 1.91, 95% CI: 1.19–3.09), cardiac death (adjusted HR: 2.00, 95% CI: 1.08–3.71), and all-cause mortality (adjusted HR: 2.78, 95% CI: 1.62–4.76). In diabetic patients, the risks were particularly pronounced when hs-cTnT levels reached ≥5 × URL. Interaction analyses showed no significant interaction between hs-cTnT levels and diabetes status regarding ischemic events (*P* = 0.78), but a significant interaction for all-cause mortality (*P* = 0.01).

**Conclusions:**

Elevated hs-cTnT levels and the presence of DM are independently associated with an increased risks of ischemic events and all-cause mortality after PCI in ACS patients. The impact of hs-cTnT on mortality is more pronounced in diabetic patients.

## 1 Introduction

In recent years, acute coronary syndrome (ACS) has emerged as a substantial healthcare burden, contributing significantly to morbidity and mortality rates (1–3). Percutaneous coronary intervention (PCI) represents an effective therapeutic strategy for managing patients diagnosed with ACS. Nonetheless, perioperative myocardial injury continues to be one of the prevalent complications associated with this procedure (4). Therefore, the implementation of an appropriate disease risk assessment model is a critical step for the early identification of patients at high risk for morbidity and mortality (5, 6). The incorporation of biomarkers to gauge the degree of myocardial damage offers a more sensitive and precise evaluation of PCI-related myocardial injury. Previous investigations have demonstrated that troponin levels are associated with perioperative myocardial injury and long-term adverse outcomes (7).

Diabetes Mellitus (DM) constitutes a significant risk factor that affects the characteristics and progression of coronary artery disease (8). Compared to individuals without DM, those with DM exhibit a higher prevalence of cardiovascular diseases (9). Recent studies have established an association between subclinical myocardial injury, as indicated by elevated levels of high-sensitivity cardiac troponin T (hs-cTnT), and hypoglycemia (10–12). This disparity suggests that it is crucial to investigate how diabetes and non-diabetes patients differ in terms of PCI-related myocardial injury (13, 14) and long-term prognosis. Nevertheless, it remains unclear whether an increase in hs-cTnT levels heightens the anticipated risk post-PCI among both DM and non-DM patients. To date, studies on this topic are rather scarce. Delving deeper into this matter can facilitate a more profound comprehension of how PCI exerts distinct effects on these two groups of patients and assist in formulating more personalized treatment regimens.

Although previous research has examined the influence of hs-cTnT levels and DM on ACS patient outcomes, our study uniquely emphasizes the comparative analysis between DM and non-DM patients. In contrast to many previous investigations that suffered from limitations due to smaller sample sizes, our large-scale analysis enhances the generalizability and robustness of our findings. To counteract these limitations, the present study aims to conduct a large-scale retrospective cohort study, which will further elucidate the roles of hs-cTnT levels and DM in ACS patients and provide more robust evidence for clinical management. By focusing on a large cohort of 14,173 consecutive ACS patients who underwent PCI at the General Hospital of the Northern Theater Command of China between March 2016 and March 2022, this study offers valuable insights that could inform more personalized and effective treatment strategies. The significance of this study is to fill the gap in the existing literature regarding the association between changes in hs-cTnT levels and prognosis in DM and non-DM patients following PCI.

## 2 Methods

### 2.1 Study population

The study cohort was derived from a prospective, real-world, single-center registry at the General Hospital of Northern Theater Command, which recruited consecutive patients who underwent PCI between March 2016 and March 2022 (15). The study population consisted of 14,173 consecutive patients diagnosed with ACS who underwent PCI. The inclusion criteria were as follows: (1) Age ≥18 years; (2) Diagnosed with ACS according to the fourth edition by the American Heart Association (16); (3) Underwent PCI; (4) Had accessible baseline and post-PCI hs-cTnT data; (5) The patients were discharged alive. The major exclusion criteria included: (1) Patients with severe comorbidities, such as end-stage renal disease or active malignancy, that may potentially impact primary outcomes; (2) Patients who experienced major adverse clinical events during hospitalization.

### 2.2 Variables

The exposure variables were the levels of hs-cTnT and the presence of DM. The diagnosis of ACS in this study was made according to the 4th Universal Definition of Myocardial Infarction (16). Specifically, the diagnostic criteria for the different types of ACS were as follows: (1) Patients were diagnosed with Unstable Angina Pectoris (UAP) if they presented with increased frequency, severity, or duration of angina attacks, no ST-segment elevation on electrocardiogram (ECG), and normal levels of cardiac injury markers (e.g., hs-cTnT) at admission; (2) Non-ST-Segment Elevation Myocardial Infarction (NSTEMI) was diagnosed if patients had elevated levels of cardiac injury markers hs-cTnT levels above the 99th percentile reference upper limit) without ST-segment elevation on ECG; (3) ST-Segment Elevation Myocardial Infarction (STEMI): Elevated levels of cardiac injury markers (high-sensitivity troponin T levels above the 99th percentile reference upper limit) with ST-segment elevation on ECG. DM was defined based on the criteria set forth by the American Diabetes Association (17). Blood samples for hs-cTnT analysis were collected at admission and again within the second day following the PCI procedure. Blood samples for hs-cTnT analysis were collected at admission and again within the second day following the PCI procedure. The hs-cTnT levels were measured using the Roche Elecsys Troponin T high-sensitivity assay on a Roche Cobas e 411 analyzer (Roche Diagnostics, Basel, Switzerland). This assay employs the electrochemiluminescence immunoassay (ECLIA) method. The lower limit of quantification for hs-cTnT was ≤0 ng/ml, and the 99th percentile upper reference limit (URL) was 0.1 ng/ml. For the analysis, we utilized the hs-cTnT levels measured on the second day after PCI to assess the association with ischemic events and mortality. The admission hs-cTnT levels were used for descriptive purposes only, to provide a baseline comparison. The primary outcome was the occurrence of ischemic events within 12 months after PCI, defined as a composite of cardiac death, non-fatal myocardial infarction (MI), and/or stroke. The secondary outcomes included all-cause mortality at 12 months and the individual components of the primary outcome. For patients with negative pre-procedural hs-TnT and post-procedural hs-TnT ≥ 5 × URL, this indicates the occurrence of periprocedural myocardial infarction (PMI) (18, 19).

### 2.3 Ethic statement

This study was approved by the local ethics committee of the General Hospital of the Northern Theater Command (Ethics Number: 2025-77) and a waiver of the requirement to obtain informed consent was provided to conduct this analysis. Written informed consent was obtained from all participating patients. The study was conducted in accordance with the principles of the Declaration of Helsinki.

### 2.4 Follow-up

To ensure the accuracy and comprehensiveness of the follow—up data. Clinical follow-up assessments were routinely conducted at 3, 6, 9, and 12 months post-procedure through phones, outpatient visits, or unscheduled readmissions by research staff. All clinical incidents were scrutinized by a clinical events committee.

### 2.5 Statistical analysis

The enrolled patients were stratified based on their DM status and the fold increase above URL of hs-cTnT. Continuous variables were summarized as mean ± standard deviation and compared using analysis of variance or the Kruskal–Wallis test, depending on data distribution. Categorical variables were expressed as frequencies and percentages, and comparisons were made using *χ*² tests or Fisher's exact tests, as appropriate. Time-to-event outcomes were analyzed using the Kaplan–Meier method and compared with the log-rank test. To assess the relationship between outcomes and hs-cTnT levels stratified by DM status, both the Cox proportional hazards regression model and restricted cubic spline analysis were employed. Variables adjusted in the multivariable model included age, gender, hypertension, previous MI, previous PCI, previous stroke, smoking status, type of ACS, anemia, eGFR, arterial access, coronary arteries treated, and number of stents. Unless otherwise specified, statistical significance was set at a two-sided *p*-value < 0.05. All statistical analyses were conducted using SAS software version 9.4 (SAS Institute).

## 3 Results

### 3.1 Study population stratification

After screening consecutive patients meeting the inclusion criteria, 14,173 patients enrolled in final analysis with a 99.3% follow-up rate at 12 months. The study population was stratified into four groups based on hs-cTnT levels and the diagnosis of DM: No DM with hs-TnT < 5 URL (*N* = 7,824); No DM with hs-TnT ≥ 5 URL (*N* = 1,971); DM with hs-TnT < 5 URL (*N* = 3,671); DM with hs-TnT ≥ 5 URL (*N* = 707). This stratification was based on the 99th percentile URL of hs-cTnT, which is 0.1 ng/ml. The stratification allowed us to assess the impact of different levels of hs-cTnT on clinical outcomes.

### 3.2 Baseline characteristics

Table 1 shows that compared with non-DM patients, DM patients with hs-cTnT levels ≥5 × URL were older (62.44 ± 10.73 years vs. 60.57 ± 10.23 years, *P* < 0.0001), had a lower proportion of males (70.01% vs. 74.87%, *P* < 0.0001), and a higher prevalence of hypertension (66.15% vs. 60.47%, *P* < 0.0001). Additionally, DM patients were more likely to have a history of MI and PCI (12.94% and 14.99%, respectively). Among DM patients with hs-cTnT levels ≥5 × URL had lower hemoglobin levels (28.01% vs. 12.87%, *P* < 0.0001). These results indicate that elevated hs-cTnT levels are closely related to adverse baseline characteristics in DM patients.

**Table 1:** Baseline characteristics of individuals stratified by levels of Hs-TnT and DM.

Group	No DM		DM		*P* value
	Non DM with hsTnT < 5 URL	Non DM with hsTnT ≥ 5 URL	DM with hsTnT < 5 URL	DM with hsTnT ≥ 5 URL	
	(*N* = 7,824)	(*N* = 1,971)	(*N* = 3,671)	(*N* = 707)	
Age, years	60.57 ± 10.23	60.70 ± 11.91	61.86 ± 9.58	62.44 ± 10.73	<.0001
Male	5,858 (74.87%)	1,543 (78.29%)	2,488 (67.77%)	495 (70.01%)	<.0001
Medical history					
Hypertension	4,729 (60.47%)	1,007 (51.09%)	2,622 (71.42%)	467 (66.15%)	<.0001
Diabetes	0 (0.00%)	0 (0.00%)	3,671 (100.00%)	707 (100.00%)	<.0001
Previous MI	1,502 (19.23%)	194 (9.85%)	884 (24.19%)	91 (12.94%)	<.0001
Previous PCI	2,096 (26.81%)	218 (11.07%)	1,271 (34.63%)	106 (14.99%)	<.0001
Previous stroke	1,071 (13.71%)	280 (14.24%)	623 (16.99%)	127 (17.96%)	<.0001
Smoking					<.0001
Never	3,257 (41.74%)	640 (32.55%)	1,836 (50.19%)	322 (45.54%)	
Active	3,316 (42.49%)	1,133 (57.63%)	1,257 (34.36%)	312 (44.13%)	
Former	1,231 (15.77%)	193 (9.82%)	565 (15.45%)	73 (10.33%)	
Type of ACS					<.0001
UA	5,575 (71.26%)	168 (8.52%)	2,713 (73.90%)	76 (10.75%)	
NSTEMI	1,285 (16.42%)	397 (20.14%)	606 (16.51%)	186 (26.31%)	
STEMI	964 (12.32%)	1,406 (71.33%)	352 (9.59%)	445 (62.94%)	
Anemia	1,007 (12.87%)	373 (18.92%)	674 (18.36%)	198 (28.01%)	<.0001
eGFR, ml/min per 1.73 m^2^	93.58 ± 21.83	85.02 ± 22.21	94.27 ± 27.51	84.82 ± 28.25	<.0001
LVEF, %	60.04 ± 7.78	53.33 ± 8.46	58.99 ± 8.64	52.47 ± 9.19	<.0001
Procedure information					
Transradial access	7,217 (92.24%)	1,774 (90.01%)	3,337 (90.90%)	638 (90.24%)	0.0025
Coronary arteries treated					
LM	513 (6.56%)	64 (3.25%)	255 (6.95%)	38 (5.37%)	<.0001
LAD	4,416 (56.44%)	957 (48.55%)	2,031 (55.33%)	340 (48.09%)	<.0001
LCX	1,996 (25.51%)	384 (19.48%)	993 (27.05%)	144 (20.37%)	<.0001
RCA	2,773 (35.44%)	770 (39.07%)	1,395 (38.00%)	300 (42.43%)	<.0001
Number of stents	1.64 ± 0.88	1.32 ± 0.77	1.72 ± 0.93	1.44 ± 0.84	<.0001
Total length of stents, mm	45.32 ± 26.30	38.26 ± 21.25	48.71 ± 27.38	42.29 ± 23.48	<.0001
Average stent diameters, mm	3.07 ± 0.80	3.06 ± 0.48	2.98 ± 0.74	3.01 ± 0.85	6.82997
Aspirin	7,722 (98.70%)	1,879 (95.33%)	3,614 (98.45%)	665 (94.06%)	<.0001
P2Y12 inhibitors					<.0001
Clopidogrel	5,432 (69.67%)	1,223 (62.53%)	2,482 (67.91%)	438 (63.02%)	
Ticagrelor	2,365 (30.33%)	733 (37.47%)	1,173 (32.09%)	257 (36.98%)	
Statins	7,344 (93.87%)	1,777 (90.16%)	3,443 (93.79%)	628 (88.83%)	<.0001
ACEI/ARB	5,088 (65.03%)	1,311 (66.51%)	2,540 (69.19%)	466 (65.91%)	0.0002
βblockers	5,302 (67.77%)	1,280 (64.94%)	2,731 (74.39%)	474 (67.04%)	<.0001

### 3.3 Clinical outcomes

The primary outcome of ischemic events at 1 year in patients with DM and hs-TnT levels ≥5 URL were significantly higher with incidences of ischemic events (5.66%), cardiac death (3.96%), MI (1.27%), and death from any cause (5.66%) compared to other groups (Table 2). Non-DM patients with hs-TnT levels ≥5 URL also had higher rates of ischemic events (3.40%) and cardiac death (1.78%). The *P* values for all outcomes were less than 0.0001, indicating statistically significant differences among the groups.

**Table 2:** Clinical outcomes of individuals stratified by levels of Hs-TnT and DM.

Outcome	Non DM		DM		*P* value
	hsTnT < 5 URL	hsTnT ≥ 5 URL	hsTnT < 5 URL	hsTnT ≥ 5 URL	
	(*N* = 7,824)	(*N* = 1,971)	(*N* = 3,671)	(*N* = 707)	
Ischemic events	109 (1.39%)	67 (3.40%)	95 (2.59%)	40 (5.66%)	<.0001
Cardiac death	51 (0.65%)	35 (1.78%)	45 (1.23%)	28 (3.96%)	<.0001
MI	30 (0.38%)	24 (1.22%)	19 (0.52%)	9 (1.27%)	<.0001
Stroke	35 (0.45%)	10 (0.51%)	35 (0.95%)	4 (0.57%)	0.0109
Death from any cause	85 (1.09%)	40 (2.03%)	55 (1.50%)	40 (5.66%)	<.0001

**Figure 1:**
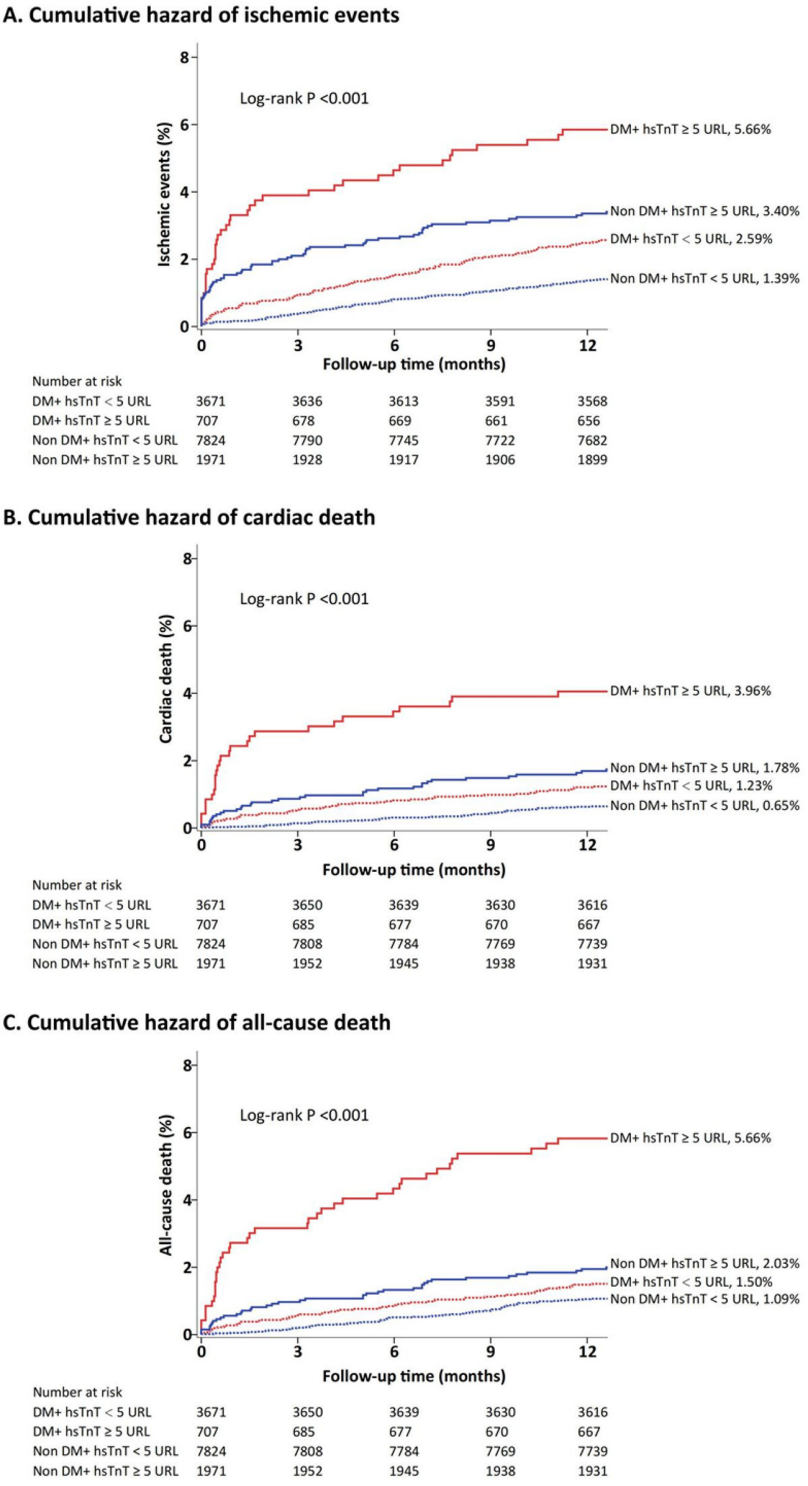
Cumulative incidence of ischemic events, cardiac death, and all-cause death stratified by hs-TnT levels.

Figure 1 demonstrates the cumulative incidence of ischemic events stratified by hs-TnT levels (1.39% vs. 2.59% vs. 3.40% vs. 5.66%, *P* < 0.001). Additionally, the cumulative incidence of cardiac death events stratified by hs-TnT levels was 0.65% vs. 1.23% vs. 1.78%vs. 3.96% (*P* < 0.001), and the cumulative incidence of all-cause death events was 1.09% vs. 1.50% vs. 2.03% vs. 5.66% (*P* < 0.001). The cumulative incidence of ischemic events, cardiac death events, and all-cause death events increased significantly with higher hs-TnT levels.

**Figure 2:**
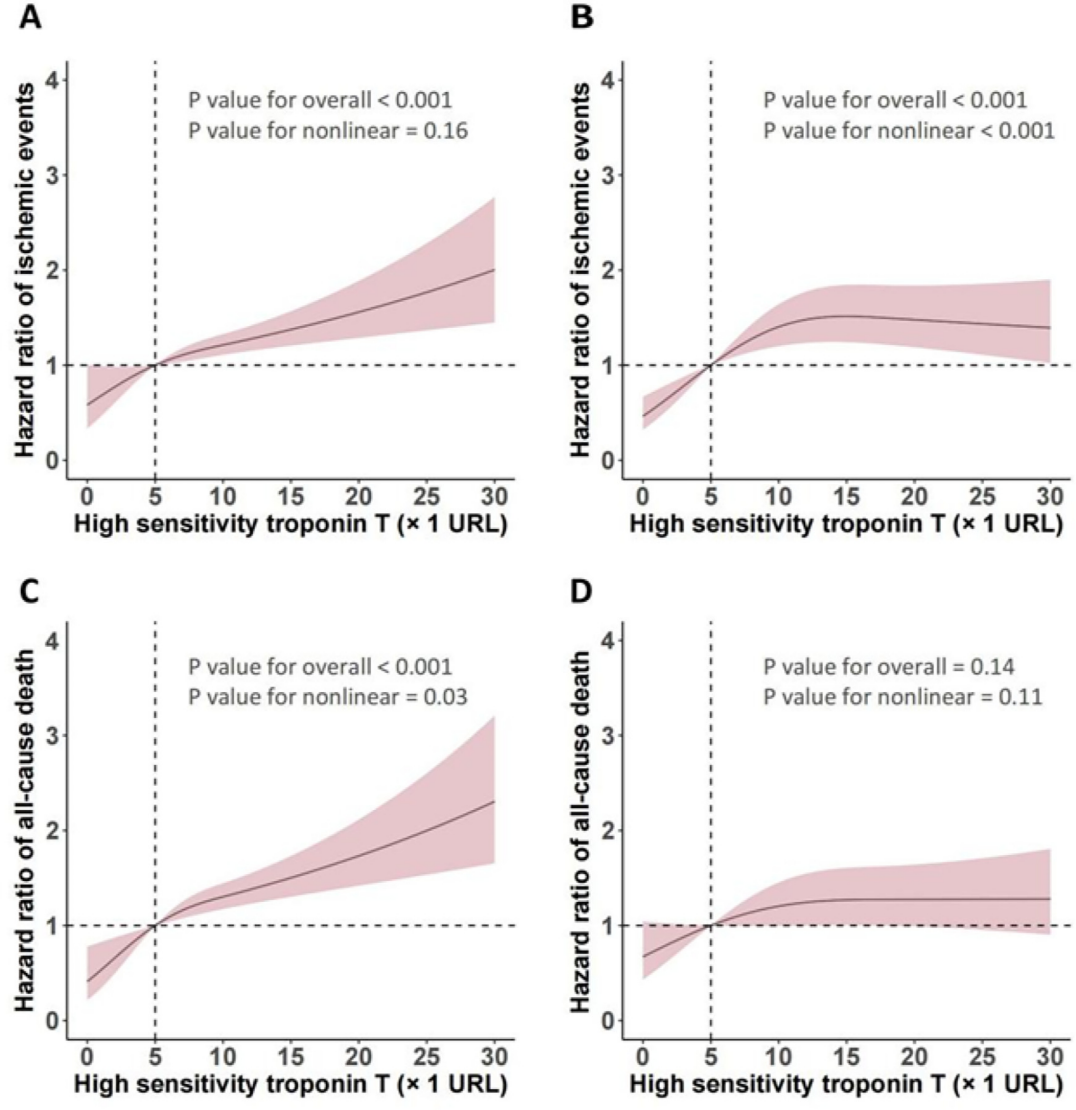
Relationship between hs-TnT levels and ischemic events as well as all-cause death analyzed by restricted cubic spline (RCS).

**Figure 3:**
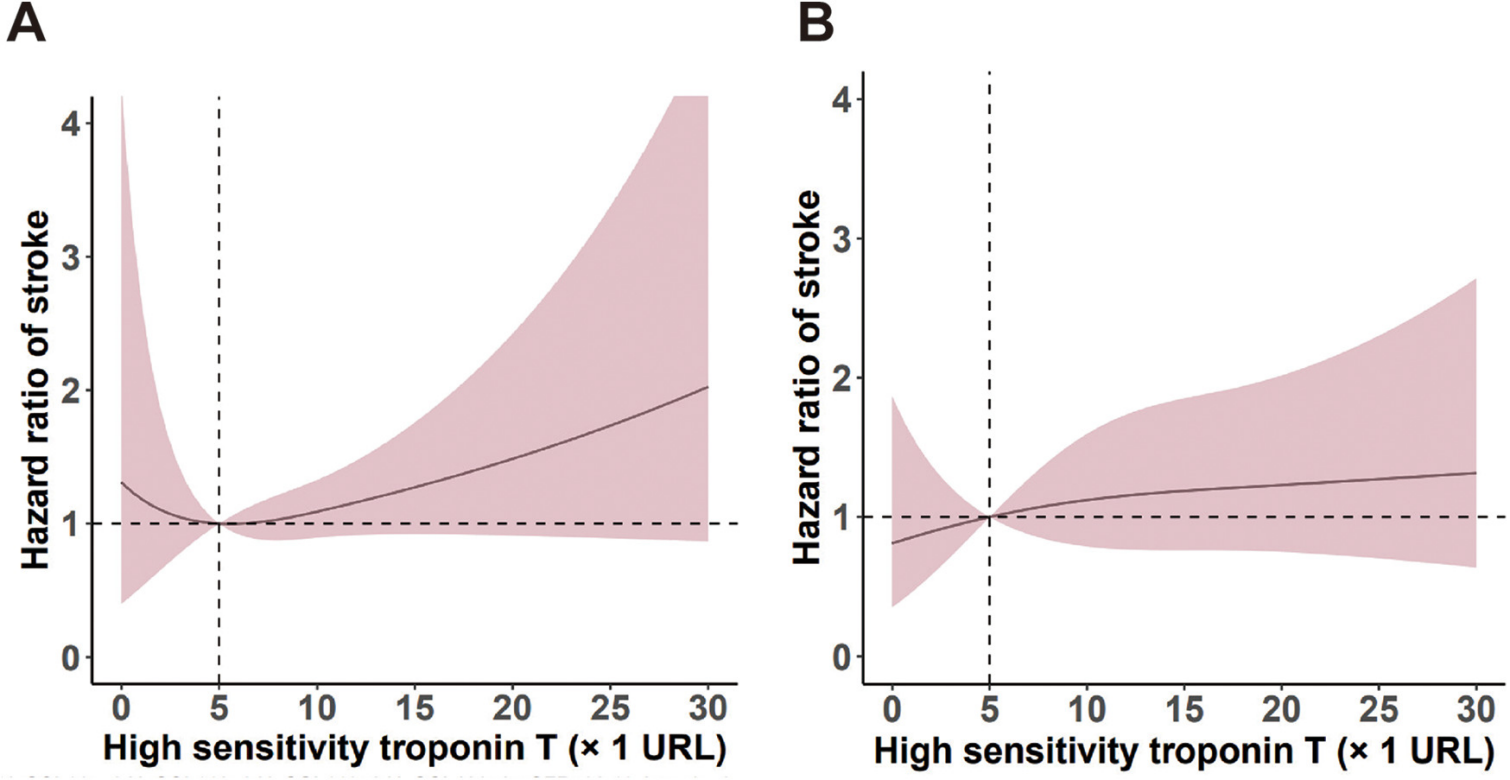
Predictive roles of hs-TnT levels for stroke events in patients with and without DM.

Using RCS (Figure 2), we identified a liner relationship between and ischemic events (*P* for non-linearity = 0.16) and an inverse J-shaped relationship between hs-TnT and non-DM in ischemic events (*P* for non-linearity < 0.001). In the DM patients, the risk of ischemic events (*P* < 0.001) and all-cause death (*P* < 0.001) manifested a slow upward trend with the increase of hsTnT, but a significant difference was still seen at the cut-off of 5 URL. RCS analysis suggests a possible linear relationship between hsTnT and all-cause death events in non-DM patients (*P* for nonlinearity = 0.11). Figure 3 shows both the cumulative incidence and the hazard ratio of stroke according to hs-cTnT levels, which illustrates the relationship between hs-cTnT levels and the risk of stroke in patients with DM. This figure illustrates the relationship between hs-cTnT levels and the risk of stroke in non-DM patients.

### 3.4 Clinical outcomes according to hs-TnT and DM status

#### 3.4.1 Cox regression analysis

DM patients with hs-TnT levels ≥5 URL had a significantly higher adjusted HR for ischemic events (HR: 1.91, 95% CI: 1.19–3.09, *P* = 0.0079, *P* for interaction = 0.7773) and all-cause death (HR: 2.78, 95% CI: 1.62–4.76, *P* = 0.0002, *P* for interaction = 0.0125) (Table 3). Non-DM patients with hs-TnT levels ≥5 URL also showed a higher adjusted HR in ischemic events (HR: 2.32, 95% CI: 1.54–3.52, *P* < 0.0001) and cardiac death (HR: 1.98, 95% CI: 1.11–3.52, *P* = 0.0207). Besides following the adjustment for baseline covariates, higher levels of hs-TnT were associated with increased incidences of ischemic events and cardiac death stratified by DM status.

**Table 3:** Cox regression analyses of Hs-TnT and DM for predicting clinical outcomes.

Outcomes	hsTnT		Adjusted hazard ratio (95% CI)	*P* value	*P* value for interaction
	<5 URL	≥5 URL			
Ischemic events					0.7773
Diabetes mellitus	2.59% (95/3,671)	5.66% (40/707)	1.91 (1.19–3.09)	0.0079	
Non diabetes mellitus	1.39% (109/7,824)	3.40% (67/1,971)	2.32 (1.54–3.52)	<0.0001	
Cardiac death					0.5808
Diabetes mellitus	1.23% (45/3,671)	3.96% (28/707)	2.00 (1.08–3.71)	0.0285	
Non diabetes mellitus	0.65% (51/7,824)	1.78% (35/1,971)	1.98 (1.11–3.52)	0.0207	
Death from any cause					0.0125
Diabetes mellitus	1.50% (55/3,671)	5.66% (40/707)	2.78 (1.62–4.76)	0.0002	
Non diabetes mellitus	1.09% (85/7,824)	2.03% (40/1,971)	1.50 (0.92–2.56)	0.107	
Stroke					
Diabetes mellitus	0.95% (35/3,671)	0.57% (4/707)	1.00 (0.91–1.10)	0.99	0.9438
Non diabetes mellitus	0.45% (35/7,824)	0.51% (10/1,971)	1.00 (0.94–1.07)	0.96	

Model adjusted for age, gender, hypertension, previous myocardial infarction, previous percutaneous coronary intervention, previous stroke, smoking, type of ACS, anemia, eGFR, arterial access, coronary arteries treated, and number of stents.

#### 3.4.2 Predictive roles of hs-TnT and DM for MACE across different ACS types

Table 4 shows the predictive roles of hs-cTnT and DM across different dignosis of ACS: UAP, NSTEMI, and STEMI. The adjusted HR and 95% CI are provided for ischemic events. For UAP, the HR for DM patients with hs-TnT ≥ 5 URL was 1.36 (95% CI: 0.42–4.37), which was not statistically significant. However, for NSTEMI and STEMI, DM patients with hs-TnT ≥ 5 URL had significantly higher HRs compared to those with hs-TnT < 5 URL (NSTEMI: HR: 1.21, 95% CI: 0.50–2.89; STEMI: HR: 2.89, 95% CI: 1.15–7.28). The *P* values for interaction were not significant for UA and NSTEMI but were significant for STEMI (*P* = 0.0246). The results indicated that among non-diabetic patients with NSTEMI, those with hs-TnT levels ≥5 × URL had a significantly higher risk of ischemic events compared to those with lower hs-TnT levels. Specifically, the adjusted hazard ratio (HR) for ischemic events was 2.23 (95% CI: 1.12–4.42, *P* = 0.0218). The results section should explicitly mention that NSTEMI in non-DM patients has predictive value.

**Table 4:** Predictive roles of hs-TnT and DM for MACE across different ACS types.

Ischemic events	hsTnT		Adjusted hazard ratio (95% CI)	*P* value	*P* value for interaction
	<5 URL	≥5 URL			
UA					0.646
Diabetes mellitus	2.54% (69/2,713)	3.95% (3/76)	1.36 (0.42–4.37)	0.6072	
Non diabetes mellitus	1.43% (80/5,575)	3.57% (6/168)	2.01 (0.86–4.69)	0.1069	
NSTEMI					0.3229
Diabetes mellitus	3.30% (20/606)	4.84% (9/186)	1.21 (0.50–2.89)	0.6727	
Non diabetes mellitus	1.56% (20/1,285)	4.28% (17/397)	2.23 (1.12–4.42)	0.0218	
STEMI					0.7685
Diabetes mellitus	1.70% (6/352)	6.29% (28/445)	2.89 (1.15–7.28)	0.0246	
Non diabetes mellitus	0.93% (9/964)	3.13% (44/1,406)	2.86 (1.34–6.14)	0.0068	

#### 3.4.3 Comparison of biomarkers in patients with DM and non-DM across different ischemic events

Table 5 compares the predictive roles of hs-cTnT and Creatine Kinase-MB (CK-MB) across different ischemic events in patients with and without DM. The adjusted HR and 95% CI are presented for ischemic events. For hs-cTnT, DM patients with levels ≥5 URL had a significantly higher HR compared to those with levels <5 URL (HR: 1.91, 95% CI: 1.19–3.09). Non-DM patients also showed a higher HR for levels ≥5 URL (HR: 2.32, 95% CI: 1.54–3.52). For CK-MB, the HRs were not significantly different between DM and non-DM patients for levels <1 URL and ≥1 URL. The *P* values for interaction were not significant for both hs-cTnT and CK-MB.

**Table 5:** Comparison of biomarkers in patients with DM and Non-DM across different ischemic events.

Outcome	Biomarker		Adjusted hazard ratio (95% CI)	*P* value	*P* value for interaction
	hsTnT < 5 URL	hsTnT ≥ 5 URL			
Ischemic events					0.7773
Diabetes mellitus	2.59% (95/3,671)	5.66% (40/707)	1.91 (1.19–3.09)	0.0079	
Non diabetes mellitus	1.39% (109/7,824)	3.40% (67/1,971)	2.32 (1.54–3.52)	<0.0001	
Outcome	Biomarker		Adjusted hazard ratio (95% CI)	*P* value	*P* value for interaction
	CKMB < 1 URL	CKMB ≥ 1 URL			
Ischemic events					0.7278
Diabetes mellitus	3.04% (122/4,011)	3.54% (13/367)	1.04 (0.59–1.86)	0.8904	
Non diabetes mellitus	1.74% (153/8,798)	2.31% (23/997)	1.23 (0.78–1.92)	0.3734	

## 4 Discussion

Our study, based on a large-scale real-world registry, explored the value of incorporating hs-cTnT and DM into the risk stratification system for ACS patients undergoing PCI in the context of contemporary therapies. The key findings are as follows: In diabetic patients, elevated hs-cTnT levels (≥5 × URL) were significantly associated with higher risks of ischemic events, cardiac death, and all-cause mortality. Specifically, compared with patients with hs-cTnT levels <1 × URL, those with hs-cTnT levels ≥5 × URL had a 1.91-fold (95% CI: 1.19–3.09) increased risk of ischemic events, a 2.00-fold (95% CI: 1.08–3.71) increased risk of cardiac death, and a 2.78-fold (95% CI: 1.62–4.76) increased risk of all-cause mortality. This highlights the prognostic value of hs-cTnT in identifying high-risk diabetic patients after PCI. Moreover, our study compared the predictive roles of hs-cTnT and CK-MB in diabetic and non-diabetic patients. The results showed that while hs-cTnT levels were significantly associated with the risk of ischemic events in diabetic patients, CK-MB levels did not show a significant difference, indicating that hs-cTnT may be a more sensitive marker for predicting ischemic events in diabetic patients. Further subgroup analyses revealed that the predictive value of hs-cTnT and DM varied among different types of ACS patients.

Even with optimal medical therapies, the recurrence rate of major adverse cardiovascular events (MACE) in ACS patients after PCI remains disappointingly high, reaching 20% in three years, and over a quarter of patients undergoing coronary interventions are diagnosed with DM (20–23). Recent research by numerous scholars highlights clinicians'growing concern for high-risk populations of such diseases (24–26). Previous studies have shown that low blood sugar levels can lead to a higher death rate among diabetic individuals (27). In HFrEF, diabetic patients exhibited elevated TnT levels compared to non-diabetic patients, whereas NT-proBNP levels were similar regardless of diabetes status (28). A significant finding of this large-scale retrospective cohort study is that the impact of hs-cTnT levels on all-cause mortality is more prominent in DM patients. Specifically, multivariable analysis showed that for every 1-fold increase in hs-cTnT levels, the risk of all-cause mortality in DM patients increased by 2.78 times (adjusted hazard ratio: 2.78, 95% CI: 1.62–4.76), highlighting the importance of diabetes status in evaluating the prognosis of ACS patients after PCI.

In this study, we employed a rigorous design, including multivariable Cox regression and restricted cubic spline analyses, to effectively address potential confounding factors. This approach not only strengthens the robustness of our findings but also provides a more nuanced understanding of the relationship between hs-cTnT levels and long-term outcomes in patients with DM.

While the findings of this study highlight the significant association between DM, elevated hs-cTnT levels, and mortality, it is essential to delve deeper into the potential biological mechanisms underlying this relationship. Hyperglycemia and chronic inflammation, both hallmarks of diabetes, may play a crucial role in amplifying the risks associated with elevated hs-cTnT levels. Elevated blood glucose levels can lead to advanced glycation end-products (AGEs) (29, 30), which contribute to oxidative stress and endothelial dysfunction. These changes may exacerbate MI and promote the release of cardiac troponins. Additionally, DM is associated with a pro-inflammatory state, characterized by elevated levels of cytokines such as TNF-α and IL-6 (31). This chronic inflammation can further impair cardiac function and increase the risk of adverse cardiovascular events. Future research should focus on elucidating these mechanisms and exploring potential therapeutic targets to mitigate the risks associated with elevated hs-cTnT levels in DM patients.

To address the comparability with prior studies, we have included additional biomarkers such as CK-MB in our analysis. As shown in Table 5, the predictive roles of hs-cTnT and CK-MB for ischemic events were compared across patients with and without diabetes mellitus. While hs-cTnT levels were significantly associated with increased risk of ischemic events in DM patients (adjusted HR: 1.91, 95% CI: 1.19–3.09), CK-MB levels did not show a significant difference between DM and non-DM patients (adjusted HR: 1.04, 95% CI: 0.59–1.86 for DM patients with CK-MB ≥ 1 URL). This suggests that hs-cTnT may be a more sensitive marker for predicting ischemic events in diabetic patients compared to CK-MB. These findings are consistent with previous studies that have demonstrated the superior sensitivity of hs-cTnT in detecting myocardial injury.

Our study confirms that elevated hs-cTnT levels and DM are associated with increased ischemic event risk post-PCI. Hs-cTnT serves as a specific indicator of heart muscle injury (32). The risk of cardiovascular ischemic events is heightened by both DM and Chronic Kidney Disease independently, as a result of the patients' pro-thrombotic and pro-inflammatory status (33, 34). Our larger cohort (14,173 patients) analyzed long-term outcomes and the interaction between hs-cTnT and DM status. Our use of restricted cubic spline analysis revealed a significant interaction between hs-cTnT and DM status, suggesting a stronger prognostic value in DM patients, possibly due to diabetes-related metabolic and inflammatory changes. Our study's larger sample size and advanced methods enhance the robustness of findings, highlighting the importance of considering DM status when evaluating hs-cTnT's prognostic value post-PCI.

The clinical value of this study lies in providing a new perspective and tool for risk assessment in patients with ACS following PCI. High-sensitivity cardiac troponins may serve as critical markers for identifying older adults at heightened risk of mortality, thereby proving instrumental in informing the clinical management of older adults diagnosed with diabetes (35). Clinical outcomes highlight that disturbances in systemic and cardiac glucose metabolism among DM patients significantly influence cardiac contractility and function, subsequently contributing to ventricular dysfunction (36). DM group exhibited elevated levels of inflammation-related biomarkers and a higher severity score, with a greater progression to severe coronary artery disease compared to non-DM group (37). The plasma concentration of epinephrine exhibited a significant reduction in individuals with diabetes compared to those without diabetes, and demonstrated a significant increase in DM subjects subsequent to both dapagliflozin (DAPA) and placebo administrations (38). Compared with previous studies, the unique contribution of this study is its large sample size and in-depth analysis of the prognostic impact in DM patients, which makes the findings more reliable. The results of this study may prompt clinicians to pay more attention to the comprehensive consideration of diabetes status and hs-cTnT levels when evaluating the post-PCI risk in ACS patients. For ACS patients with diabetes, especially those with significantly elevated hs-cTnT levels, it is recommended to enhance postoperative monitoring and management, and optimize secondary prevention measures for cardiovascular diseases, such as intensified antiplatelet therapy, blood glucose control, and lifestyle interventions.

Moreover, the study's focus on Chinese patients means that the findings may not be fully applicable to other ethnic groups. As an observational study, this research can only detect associations rather than prove causality. Although we adjusted for numerous potential confounders in the multivariable models, unmeasured confounding factors may still influence the results. Additionally, the inclusion and exclusion criteria of the study may further limit the extrapolation of the findings. For instance, patients with certain severe comorbidities were excluded from this study, meaning that the findings cannot be directly applied to these patient populations. Future research should consider including more diverse patient populations to enhance the representativeness and generalizability of the results.

In summary, our study underscores the need for future interventional studies to elucidate the causal relationships between elevated hs-cTnT levels, diabetes mellitus, and adverse cardiovascular outcomes, while highlighting the importance of personalized post-PCI monitoring and management strategies in DM patients with elevated hs-cTnT levels to improve their long-term outcomes.

## 5 Study limitations

Our study has several limitations. First, the limitations of this study primarily stem from its single-center design, which to some extent restricts the generalizability of the findings and necessitates validation through additional similar studies. Second, the study relied solely on hs-cTnT for evaluating MI, and the consistency of other biomarkers such as Troponin I, High-sensitivity cardiac Troponin I and Creatine Kinase in patients with diabetes remains to be further evaluated. Third, the study's focus on Chinese patients means that the findings may not be fully applicable to other ethnic groups. As an observational study, this research can only detect associations rather than prove causality. Although we adjusted for numerous potential confounders in the multivariable models, unmeasured confounding factors may still influence the results.

## 6 Conclusions

In patients with ACS who undergo PCI, elevated hs-cTnT levels and the presence of DM are each independently linked to a heightened likelihood of ischemic events and mortality. Specifically, the influence of hs-cTnT on mortality is more significant among DM patients.

## Data Availability

The original contributions presented in the study are included in the article/Supplementary Material, further inquiries can be directed to the corresponding authors.

## References

[1] Ma LY Chen WW Gao RL Liu LS Zhu ML Wang YJ. China cardiovascular diseases report 2018: an updated summary. J Geriatr Cardiol. (2020) 17:1–8. 10.11909/j.issn.1671-5411.2020.01.001 32133031 PMC7008101

[2] You SC Rho Y Bikdeli B Kim J Siapos A Weaver J Association of ticagrelor vs. clopidogrel with net adverse clinical events in patients with acute coronary syndrome undergoing percutaneous coronary intervention. JAMA. (2020) 324:1640–50. 10.1001/jama.2020.16167 33107944 PMC7592033

[3] Huang M Li D Li L Wang Y Zhang L Chen Y Factors predicting the occurrence of net adverse clinical and cerebral events in patients with acute coronary syndrome treated with clopidogrel or ticagrelor in combination with aspirin: a real-world study. Ann Transl Med. (2022) 10(2):98. 10.21037/atm-21-7038 35282096 PMC8848390

[4] Bulluck H Paradies V Barbato E Baumbach A Bøtker HE Capodanno D Prognostically relevant periprocedural myocardial injury and infarction associated with percutaneous coronary interventions: a consensus document of the ESC working group on cellular biology of the heart and European association of percutaneous cardiovascular interventions (EAPCI). Eur Heart J. (2021) 42(27):2630–42. 10.1093/eurheartj/ehab271 34059914 PMC8282317

[5] Lin H Xue Y Chen K Zhong S Chen L. Acute coronary syndrome risk prediction based on gradient boosted tree feature selection and recursive feature elimination: a dataset-specific modeling study. PLoS One. (2022) 17(11):e0278217. 10.1371/journal.pone.0278217 36445881 PMC9707772

[6] Ramzan I Ardavani A Vanweert F Mellett A Atherton PJ Idris I. The association between circulating branched chain amino acids and the temporal risk of developing type 2 diabetes mellitus: a systematic review & meta-analysis. Nutrients. (2022) 14(20):4411. 10.3390/nu14204411 36297095 PMC9610746

[7] Everett BM Moorthy MV Tikkanen JT Cook NR Albert CM. Markers of myocardial stress, myocardial injury, and subclinical inflammation and the risk of sudden death. Circulation. (2020) 142(12):1148–58. 10.1161/CIRCULATIONAHA.120.046947 32700639 PMC7995996

[8] Merino J Jablonski KA Mercader JM Kahn SE Chen L Harden M Interaction between type 2 diabetes prevention strategies and genetic determinants of coronary artery disease on cardiometabolic risk factors. Diabetes. (2020) 69(1):112–20. 10.2337/db19-0097 31636172 PMC6925585

[9] Zhao R Sun Y Zhang Y Wang W Wang S Wang C Distinguishable immunologic characteristics of COVID-19 patients with comorbid type 2 diabetes compared with nondiabetic individuals. Mediators Inflamm. (2020) 2020:6914878. 10.1155/2020/6914878 33061829 PMC7542493

[10] Lee AK McEvoy JW Hoogeveen RC Ballantyne CM Selvin E. Severe hypoglycemia and elevated high-sensitivity cardiac troponin T in older adults with diabetes: the ARIC study. J Am Coll Cardiol. (2016) 68:1370–1. 10.1016/j.jacc.2016.06.049 27634129 PMC5084452

[11] Rezende PC Everett BM Brooks MM Vlachos H Orchard TJ Frye RL Hypoglycemia and elevated troponin in patients with diabetes and coronary artery disease. J Am Coll Cardiol. (2018) 72:1778–86. 10.1016/j.jacc.2018.07.067 30286920

[12] Echouffo-Tcheugui JB Daya N Lee AK Tang O Ndumele CE Windham BG Severe hypoglycemia, cardiac structure and function, and risk of cardiovascular events among older adults with diabetes. Diabetes Care. (2021) 44(1):248–54. 10.2337/dc20-0552 33199469 PMC7783928

[13] Khin ET Aung MN Ueno S Ahmad I Latt TS Moolphate S Social support between diabetes patients and non-diabetes persons in yangon, Myanmar: a study applying ENRICHD social support instrument. Int J Environ Res Public Health. (2021) 18(14):7302. 10.3390/ijerph18147302 34299754 PMC8303506

[14] Silvain J Zeitouni M Paradies V Zheng HL Ndrepepa G Cavallini C Procedural myocardial injury, infarction and mortality in patients undergoing elective PCI: a pooled analysis of patient-level data. Eur Heart J. (2021) 42(4):323–34. Erratum in: Eur Heart J. 2021 April 7;42(14):1443. doi: 10.1093/eurheartj/ehab073. 10.1093/eurheartj/ehaa885 33257958 PMC7850039

[15] Qiu M Na K Qi Z Zhou H Li P Xu K Contemporary use of ticagrelor vs. clopidogrel in patients with acute coronary syndrome undergoing percutaneous coronary intervention: a GRACE risk score stratiﬁcation-based analysis in a large-scale, real-world study from China. Mayo Clin Proc. (2023) 98(07):1021–32. 10.1016/j.mayocp.2023.02.004 37419570

[16] Rao SV O'Donoghue ML Ruel M Rab T Tamis-Holland JE Alexander JH 2025 ACC/AHA/ACEP/NAEMSP/SCAI guideline for the management of patients with acute coronary syndromes: a report of the American college of cardiology/American heart association joint committee on clinical practice guidelines. Circulation. (2025) 151(13):e771–862. Erratum in: Circulation. 2025 Apr; 151(13):e865. doi: 10.1161/CIR.0000000000001328. PMID: 40014670. 10.1161/CIR.0000000000001309 40014670

[17] American Diabetes Association Professional Practice Committee. 2. Classification and diagnosis of diabetes: standards of medical care in diabetes-2022. Diabetes Care. (2022) 45(Suppl 1):S17–38. 10.2337/dc22-S002 34964875

[18] Thygesen K Alpert JS Jaffe AS Chaitman BR Bax JJ Morrow DA Fourth universal definition of myocardial infarction (2018). Eur Heart J. (2019) 40:237–69. 10.1093/eurheartj/ehy462 30165617

[19] Thygesen K Alpert JS Jaffe AS Chaitman BR Bax JJ Morrow DA Fourth universal definition of myocardial infarction (2018). Glob Heart. (2018) 13(4):305–38. 10.1016/j.gheart.2018.08.004 30154043

[20] Kalkman DN Aquino M Claessen BE Baber U Guedeney P Sorrentino S Residualinflamma tory risk and the impact on clinical outcomes in patients after percutaneous coronary interventions. Eur Heart J. (2018) 39(46):4101–8. 10.1093/eurheartj/ehy633 30358832

[21] Stone GW Maehara A Lansky AJ de Bruyne B Cristea E Mintz GS Aprospectivenatural-history study of coronary atherosclerosis. N Engl J Med. (2011) 364(3):226–35. 10.1056/NEJMoa1002358 21247313

[22] Kaura A Hartley A Panoulas V Glampson B Shah ASV Davies J Mortality risk prediction of high-sensitivity C-reactive protein in suspected acute coronary syndrome: a cohort study. PLoS Med. (2022) 19(2):e1003911. 10.1371/journal.pmed.1003911 35192610 PMC8863282

[23] Hu DY Pan CY Yu JM. The relationship between coronary artery disease and abnormal glucose regulation in China: the China heart survey. Eur Heart J. (2006) 27(21):2573–79. 10.1093/eurheartj/ehl207 16984927

[24] Szarek M Bittner VA Aylward P Baccara-Dinet M Bhatt DL Diaz R Lipoprotein(a) lowering by alirocumab reduces the total burden of cardiovascular events independent of low-density lipoprotein cholesterol lowering: ODYSSEY OUTCOMES trial. Eur Heart J. (2020) 41(44):4245–55. 10.1093/eurheartj/ehaa649 33051646 PMC7724642

[25] Dolejsi T Delgobo M Schuetz T Tortola L Heinze KG Hofmann U Adult T-cells impair neonatal cardiac regeneration. Eur Heart J. (2022) 43(28):2698–709. 10.1093/eurheartj/ehac153 35417553 PMC9300388

[26] Ali MK McKeever Bullard K Imperatore G Benoit SR Rolka DB Albright AL Reach and use of diabetes prevention services in the United States, 2016–2017. JAMA Netw Open. (2019) 2(5):e193160. 10.1001/jamanetworkopen.2019.3160 31074808 PMC6512285

[27] Pratiwi C Mokoagow MI Made Kshanti IA Soewondo P. The risk factors of inpatient hypoglycemia: a systematic review. Heliyon. (2020) 6(5):e03913. 10.1016/j.heliyon.2020.e03913 32420485 PMC7218453

[28] Rørth R Jhund PS Kristensen SL Desai AS Køber L Rouleau JL The prognostic value of troponin T and N-terminal pro B-type natriuretic peptide, alone and in combination, in heart failure patients with and without diabetes. Eur J Heart Fail. (2019) 21(1):40–9. 10.1002/ejhf.1359 30537261 PMC6607514

[29] Wang N Liu X Tang Z Wei X Dong H Liu Y Increased BMSC exosomal miR-140-3p alleviates bone degradation and promotes bone restoration by targeting Plxnb1 in diabetic rats. J Nanobiotechnology. (2022) 20(1):97. 10.1186/s12951-022-01267-2 35236339 PMC8889728

[30] Xu Z Qi X Bao M Zhou T Shi J Xu Z Biomineralization inspired 3D printed bioactive glass nanocomposite scaffolds orchestrate diabetic bone regeneration by remodeling micromilieu. Bioact Mater. (2023) 25:239–55. 10.1016/j.bioactmat.2023.01.024 36817824 PMC9929491

[31] de Marañón AM Díaz-Pozo P Canet F Díaz-Morales N Abad-Jiménez Z López-Domènech S Metformin modulates mitochondrial function and mitophagy in peripheral blood mononuclear cells from type 2 diabetic patients. Redox Biol. (2022) 53:102342. 10.1016/j.redox.2022.102342 35605453 PMC9124713

[32] Lee SH Cho I You SC Cha MJ Chang JS Kim WD Cancer therapy-related cardiac dysfunction in patients treated with a combination of an immune checkpoint inhibitor and doxorubicin. Cancers (Basel). (2022) 14(9):2320. 10.3390/cancers14092320 35565449 PMC9100163

[33] Capodanno D Angiolillo DJ. Antithrombotic therapy in patients with chronic kidney disease. Circulation. (2012) 125(21):2649–61. 10.1161/CIRCULATIONAHA.111.084996 22644369

[34] Gao C Tomaniak M Takahashi K Kawashima H Wang R Hara H Ticagrelor monotherapy in patients with concomitant diabetes mellitus and chronic kidney disease: a *post hoc* analysis of the GLOBAL LEADERS trial. Cardiovasc Diabetol. (2020) 19(1):179. 10.1186/s12933-020-01153-x 33066794 PMC7568378

[35] Tang O Daya N Matsushita K Coresh J Sharrett AR Hoogeveen R Performance of high-sensitivity cardiac troponin assays to reflect comorbidity burden and improve mortality risk stratification in older adults with diabetes. Diabetes Care. (2020) 43(6):1200–8. 10.2337/dc19-2043 32161049 PMC7245347

[36] Toma A Stähli BE Gick M Colmsee H Gebhard C Mashayekhi K Long-term follow-up of patients with previous coronary artery bypass grafting undergoing percutaneous coronary intervention for chronic total occlusion. Am J Cardiol. (2016) 118:1641–6. 10.1016/j.amjcard.2016.08.038 27692593

[37] Chung SM Lee YY Ha E Yoon JS Won KC Lee HW The risk of diabetes on clinical outcomes in patients with coronavirus disease 2019: a retrospective cohort study. Diabetes Metab J. (2020) 44(3):405–13. 10.4093/dmj.2020.0105 32602272 PMC7332325

[38] Solis-Herrera C Daniele G Alatrach M Agyin C Triplitt C Adams J Increase in endogenous glucose production with SGLT2 inhibition is unchanged by renal denervation and correlates strongly with the increase in urinary glucose excretion. Diabetes Care. (2020) 43(5):1065–9. 10.2337/dc19-2177 32144165 PMC7171949

